# MiGut: A scalable in vitro platform for simulating the human gut microbiome—Development, validation and simulation of antibiotic‐induced dysbiosis

**DOI:** 10.1111/1751-7915.14259

**Published:** 2023-04-10

**Authors:** William A. Davis Birch, Ines B. Moura, Duncan J. Ewin, Mark H. Wilcox, Anthony M. Buckley, Peter R. Culmer, Nikil Kapur

**Affiliations:** ^1^ School of Mechanical Engineering University of Leeds Woodhouse Lane Leeds LS2 9JT UK; ^2^ Healthcare‐Associated Infections Group Leeds Institute of Medical Research, Faculty of Medicine and Health, University of Leeds Leeds LS2 9JT UK; ^3^ Microbiology Leeds Teaching Hospitals NHS Trust, Old Medical School, Leeds General Infirmary Leeds LS1 3EX UK; ^4^ Microbiome and Nutritional Science Group, Faculty of Food Science and Nutrition, School of Food Science University of Leeds Leeds LS2 9JT UK

## Abstract

In vitro models of the human colon have been used extensively in understanding the human gut microbiome (GM) and evaluating how internal and external factors affect the residing bacterial populations. Such models have been shown to be highly predictive of in vivo outcomes and have a number of advantages over animal models. The complexity required by in vitro models to closely mimic the physiology of the colon poses practical limits on their scalability. The scalable Mini Gut (MiGut) platform presented in this paper allows considerable expansion of model replicates and enables complex study design, without compromising on in vivo reflectiveness as is often the case with other model systems. MiGut has been benchmarked against a validated gut model in a demanding 9‐week study. MiGut showed excellent repeatability between model replicates and results were consistent with those of the benchmark system. The novel technology presented in this paper makes it conceivable that tens of models could be run simultaneously, allowing complex microbiome‐xenobiotic interactions to be explored in far greater detail, with minimal added resources or complexity. This platform expands the capacity to generate clinically relevant data to support our understanding of the cause‐effect relationships that govern the GM.

## Graphical abstract

In vitro models of the human colon have been used extensively in developing an understanding of the human gut microbiome, but current technologies are extremely complex and have limited throughput. MiGut is a novel platform which addresses these shortcomings, allowing for considerable expansion of model runs without compromising on in vivo reflectiveness. The technology has been validated in a demanding 9‐week study where dysbiosis was simulated in vitro and has been benchmarked against a previously validated and extremely well understood triple‐stage gut model system.

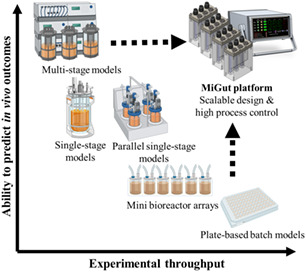

## INTRODUCTION

The gut microbiome (GM) is one of the most populous and diverse ecosystems of microorganisms found in the human body (Ursell et al., 2012) and plays a critical role in host health and disease (Cani, 2018; Kho & Lal, 2018; Marchesi et al., 2016) with disruptions to the intestinal microbiome being correlated with a myriad of health conditions (Durack & Lynch, 2019; Manor et al., 2020). Historically, there has been a huge reliance on animal (in particular mouse) models when studying the GM. However, there are physiological differences between animal and human gastrointestinal tracts, as well as compositional and functional differences of the residing microbiota which must be accounted for (Hugenholtz & de Vos, 2018; Treuting et al., 2017). Additionally, in vivo studies require ethical considerations and there is a general need to reduce the number of animals used in research. These limitations can be addressed by in vitro models designed to mimic human physiology; they allow spatial and longitudinal sampling, have fewer ethical constraints and offer good control over physiological parameters, thus improving reproducibility.

**FIGURE 1:**
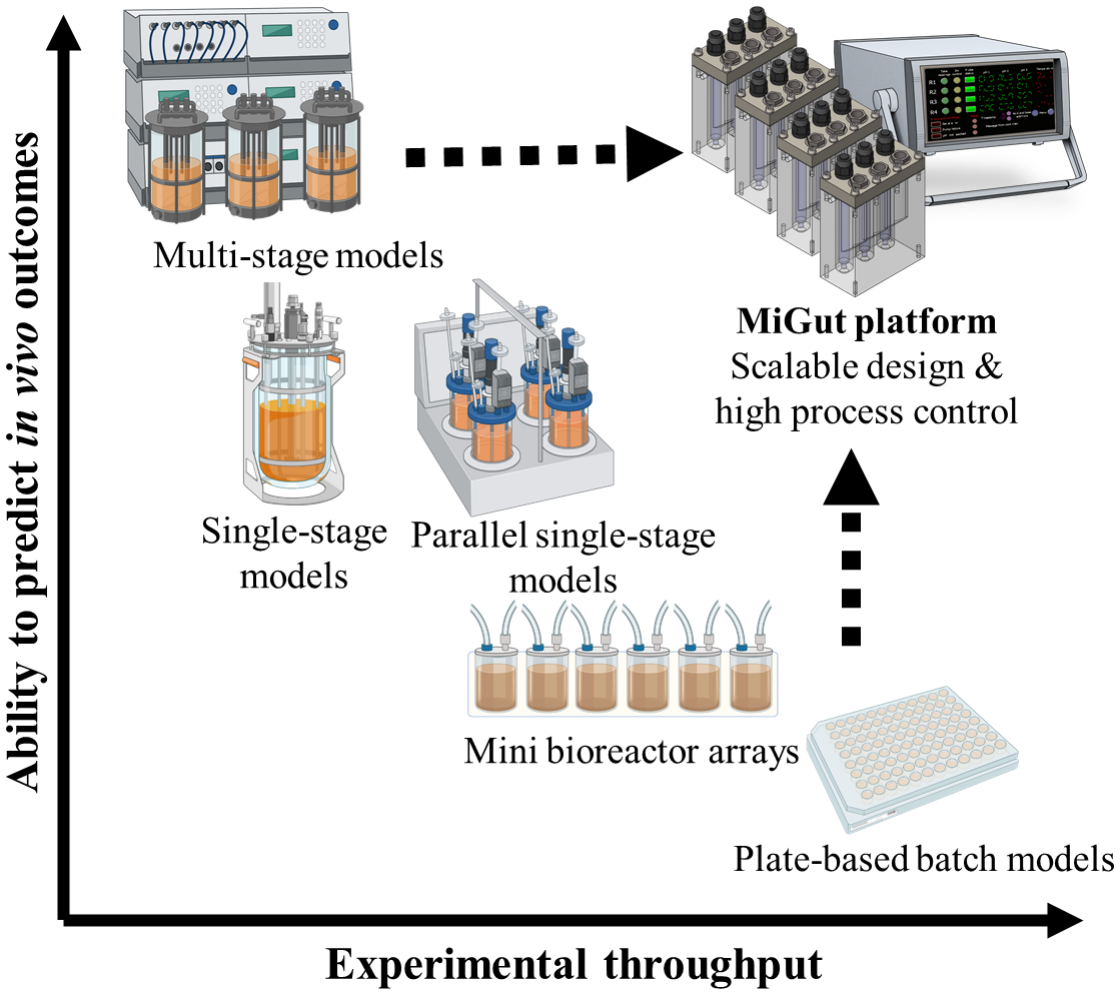
Balance of throughput and depth of process information for a range of bioreactor models. Our work targets the top right quadrant. Created with BioRender.com.

There exists a range of in vitro intestinal models, with varying levels of complexity (Roupar et al., 2021; Venema & van den Abbeele, 2013). The simplest and most easily scaled consist of batch fermentations of faecal samples (Gibbons et al., 2022; Gurry et al., 2021). Although useful for initial screening studies, these lack the physiological relevance of more complex models and are limited by short run times (<12 h). Longitudinal studies can be achieved through a continuous nutrient feed, as is the case with a number of single‐stage models in the literature (McDonald et al., 2013; Yu et al., 2021). The relatively low complexity of these models means they can easily be scaled up: for example, the MiniBioReactor Array can operate up to 48 reactors in parallel to study *Clostridioides difficile* physiology and pathogenesis (Auchtung et al., 2015). However, single‐stage models do not recapture the spatial bacterial differentiation present in the colon, making them less clinically reflective. By arranging multiple vessels in series, it is possible to simulate different colonic environments, as has been demonstrated with various gut model systems in the literature (SHIME: Van de Wiele et al., 2015; TIM: Minekus, 2015; PolyFermS: Zihler Berner et al., 2013; EnteroMix: Mäkivuokko et al., 2006). A triple‐stage chemostat model (Supporting Information, Section 2) based on the original work by Gibson et al. (1988) has been extensively used by our team for over two decades to investigate the effects of antibiotics on the GM and the pathogenesis of infections caused, for example, by *C. difficile* and multi‐drug resistant Gram‐negative bacteria (Begum et al., 2020; Best et al., 2012; Buckley, Altringham, et al., 2021; Buckley, Moura, Altringham, et al., 2021; Crowther et al., 2016; Harris et al., 2021; Moura et al., 2019, 2022; Rooney et al., 2019). This human gut model (HGM) has been previously validated against human gut content of sudden death victims, with samples taken from the proximal, medial and distal colonic regions closely matching the microbial in vitro populations in vessels 1, 2 and 3 of the triple‐stage model, respectively (Macfarlane et al., 1998). Although multi‐stage models have clear advantages in being clinically reflective, the additional complexity makes their assembly and running difficult and resource‐consuming, with large costs and space requirements (Payne et al., 2012). As a result, experimental replicates are impractical, which limits the complexity of studies that can be performed. Often, there is a trade‐off between process information/control and throughput, as illustrated in Figure 1. There are currently no multi‐stage gut model systems which adequately simulate the colonic environment while allowing for increased experimental throughput.

The Mini Gut (MiGut) platform builds upon current in vitro technologies to create a scalable colonic simulator with increased experimental throughput. Unlike other scalable systems, MiGut remains clinically reflective by retaining all the features of a standard triple‐stage system. It also takes advantage of high‐throughput molecular analysis techniques such as real‐time PCR (rtPCR) to overcome time‐consuming culture‐based methods which have been historically used with triple‐stage models (Moura et al., 2020). An initial study was performed to demonstrate how well MiGut recaptures different faecal inocula, after which the system was tested in a second 9‐week study to show (i) the platform can support a complex human microbiome over an extended period; (ii) the microbial composition within multiple MiGut models behaves consistently when exposed to external stressors, such as multiple antibiotics; (iii) the results of the model are directly comparable to those of the clinically reflective HGM.

## EXPERIMENTAL PROCEDURES

### Set‐up of the colonic models

For all studies, MiGut models were set up to mimic the HGM‐controlled conditions. Temperature and pH were identical, and the flow rate of growth medium into V1 was scaled to maintain the same retention time (48 h) considering the reduction in volume from 300 to 45 mL per vessel. Growth media consists of magnesium sulphate (0.01 g/L), calcium chloride (0.01 g/L), sodium chloride (0.1 g/L), di‐potassium monohydrogen phosphate (0.04 g/L) potassium di‐hydrogen phosphate (0.04 g/L), sodium hydrogen carbonate (2.0 g/L), haemin (0.005 g/L), cysteine HCL (0.5 g/L), bile salts (0.5 g/L), arabinogalactan (1.0 g/L), tween 80 (2 mL/L), pectin (2.0 g/L), starch (3.0 g/L), vitamin K1 (10 μL/L), peptone water (2.0 g/L), yeast extract (2.0 g/L), chenodeoxycholic acid (0.25 mg/L), lithocholic acid (0.017 mg/L), mucin (2 g/L), glucose (0.4 g/L) and resazurin anaerobic indicator (0.005 g/L), as previously described in the literature (Buckley, Moura, Arai, et al., 2021) and the Supporting Information (Section 3). The gut model fluid was continually sparged with nitrogen, which also acted as a way of mixing ‐ the internal vessel geometry is designed to resemble that of a bubble column reactor and promotes good mixing of the fluid. The HGM in the 9‐week study was assembled as previously described (Moura et al., 2020) and further outlined in the Supporting Information (Section 3).

### Preparation of faecal slurry

Each of the faecal slurries was prepared by pooling faecal samples from five healthy volunteers without history of antibiotic therapy or prescription of proton pump inhibitors in the 3 months prior to sample collection, as previously described (Moura et al., 2022; Roberts et al., 2020). Samples were homogenised with phosphate‐buffered saline to a 1:10 weight ratio (Buckley, Moura, Arai, et al., 2021). All vessels were filled up to 50% of their total volume with the same faecal slurry. Media flow was started immediately after inoculation, and pH control was turned on after 24 h, once all vessels had reached their full volume.

**FIGURE 2:**
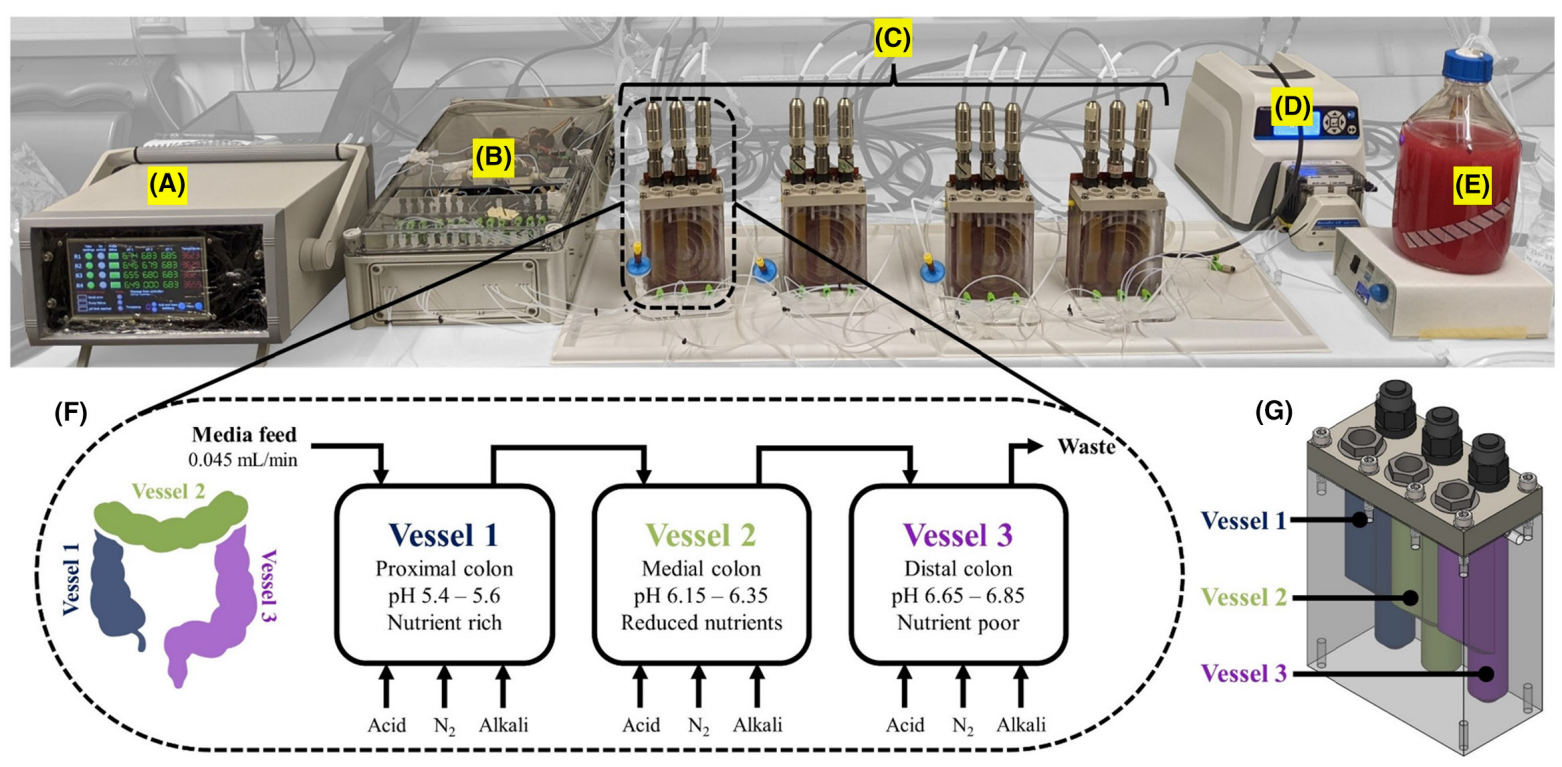
Details of the MiGut platform and model. (A) Central controller, (B) pumping unit, (C) four triple‐stage gut models each capable of running a unique set of conditions, (D) peristaltic pump for media addition, (E) nutrient‐rich media, (F) details of the flow within the reactor, (G) 3D render of reactor unit, with colour‐coded vessels.

### Experimental design

In the preliminary study comparing equilibrium to faecal slurry, four separate runs were performed‐each run lasted 2 weeks, Samples were taken from the faecal slurry before instillation in the models, and of the contents of V3 at the end of the 2‐week period.

In the subsequent 9‐week study, one HGM and one MiGut platform of 4 triple‐stage models were run in parallel. Following inoculation, bacterial populations were allowed to stabilise for 2 weeks (equilibration) and subsequently exposed to antibiotics. Each model was dosed with amoxicillin (24 mg/mL, 2× daily), ciprofloxacin (278 mg/mL, 3× daily) and piperacillin/tazobactam (828 mg/mL, 2× daily). Antibiotics were instilled only in V1 of each model and in the order described. Each antibiotic treatment was performed for 5 days, with a 2‐day recovery/washout period between them. These drugs were chosen based on an internal analysis of clinical patient data at the Leeds Teaching Hospital Trust showing these were some of the most frequently prescribed antibiotics. Furthermore, the antibiotic concentrations used are reflective of the drug values found in human colon when a patient is prescribed each antibiotic. Bacterial populations were left undisturbed for a further 4 weeks after antibiotic dosing. This experimental timeline allowed for performance under steady state to be evaluated, as well as the response to antibiotics.

### 16S rRNA sequencing analysis

Data for the PCoA plot were created with 16S rRNA gene sequencing of four testing runs. DNA was extracted from 1 mL of bioreactor fluid from V3. Briefly, samples were centrifuged (15,000 rpm, 20 min, 4°C), supernatants were discarded, and cell pellets were stored at −80°C for up to 48 h until processing. Following cell disruption using a tissue lyser, DNA extraction was performed using the DNeasy 96 PowerSoil Pro QIAcube HT Kit (Qiagen).

Briefly, 16S rRNA V4 fragments were amplified, cleaned and checked using gel electrophoresis before running a PCR for addition of the index sequences. The fragments were then cleaned, quantified, normalised and sequenced on a MiSeq (Illumina) using a 2 × 250 bp paired‐end reads cycle. Demultiplexed FASTQ files were trimmed using cutadapt (Martin, 2011) and filtered following procedures from the MOTHUR package (v.1.41.3). Unique sequences were aligned against tailor‐made reference generated from SILVA SEED database (v. 132). OTUs were identified by clustering (0.5 UniFrac distance) the sequences and were assigned the consensus taxonomy information with label = 0.03.

Taxonomic analysis is represented as mean per cent abundance from four technical replicates. The distance matrix based on thetayc approach was used for PCoAs analysis and visualisation for each group of samples, based on four technical replicates. Full details can be found in the Supporting Information (Section 5).

### Bacterial sampling and quantification

Each model was sampled thrice weekly, by collecting 1 mL of fluid from each vessel. DNA was extracted as described above and stored at −80°C. DNA extracts were normalised to 5 ng/μL and analysed by rtPCR against a plasmid curve to calculate bacterial concentrations for eight key bacterial populations (Eubacteria, *Bacteroides* spp., *Bifidobacteria*, *Clostridium coccoides* group, *Clostridium leptum* group, Enterobacteriaceae, *Lactobacillus* spp. and *Prevotella* spp.), as previously described (Moura et al., 2020). Primer sequence and rtPCR conditions are detailed in the Supporting Information (Section 4).

Data were converted to a logarithmic scale to allow for normal distribution. Figures generated using the rtPCR data give an overview of population dynamics within in vitro gut models and allow comparison with historical data collected from culture‐based methods (Moura et al., 2020).

**FIGURE 3:**
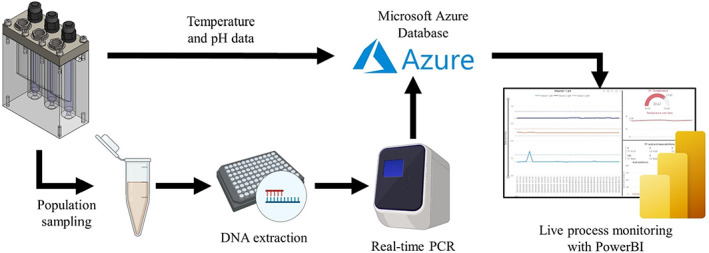
Dataflow for MiGut. Online process data (pH, temperature, acid/base additions) streamed via the cloud for remote process logging and monitoring. Population sampling from PCR giving temporal and spatial sampling. Created with BioRender.com.

**FIGURE 4:**
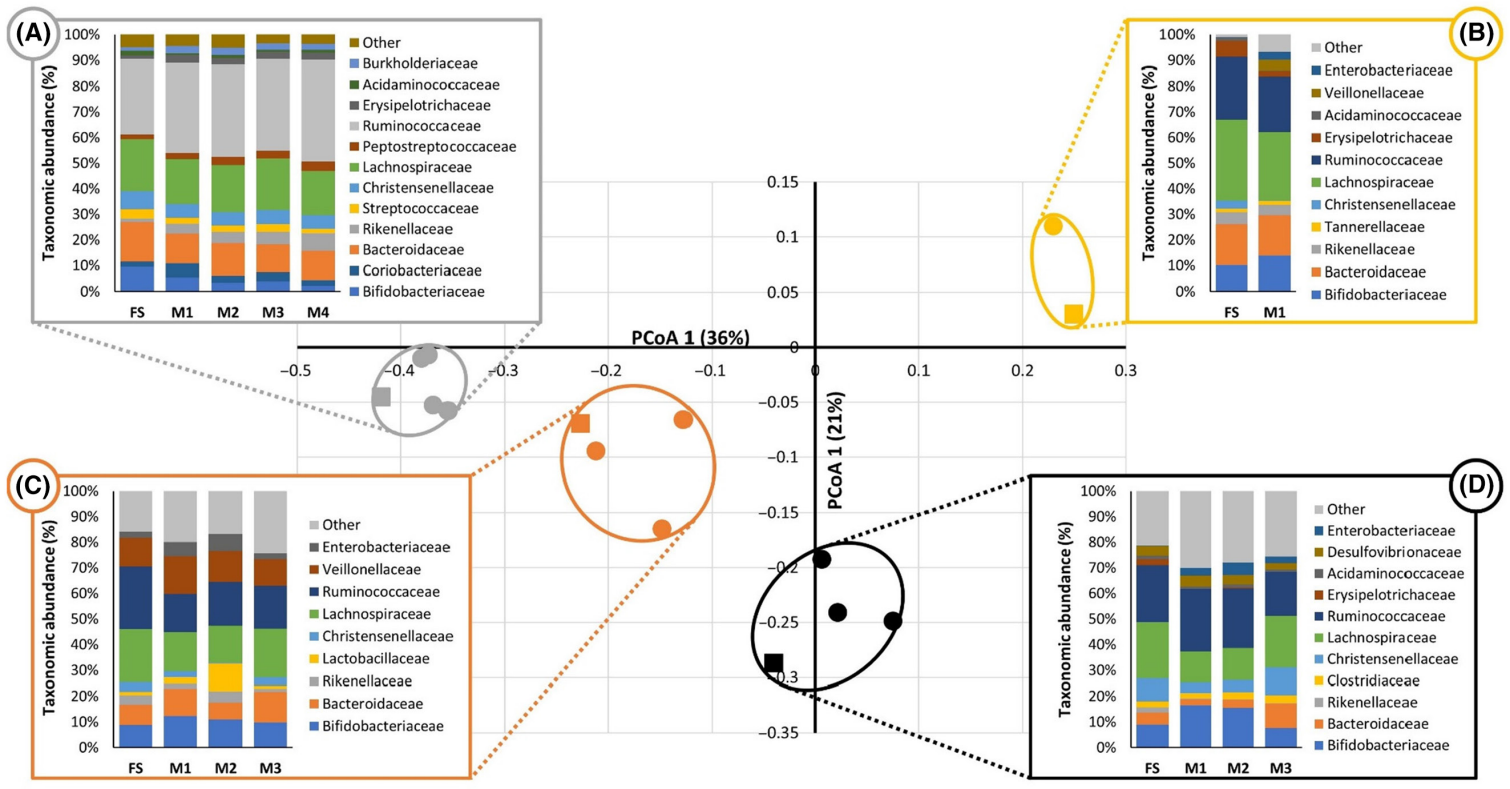
Principal coordinate analysis plot (derived from the thetayc distance matrix) showing four different faecal slurries (squares) and the microbial ecology in the corresponding models (circles; each circle represents an individual model). The taxonomic profile for the faecal slurry (FS) and the individual models (M1–3) are shown in each coloured box (A–D), with the colours matching the independent experiments shown in the PCoA plot.

**FIGURE 5:**
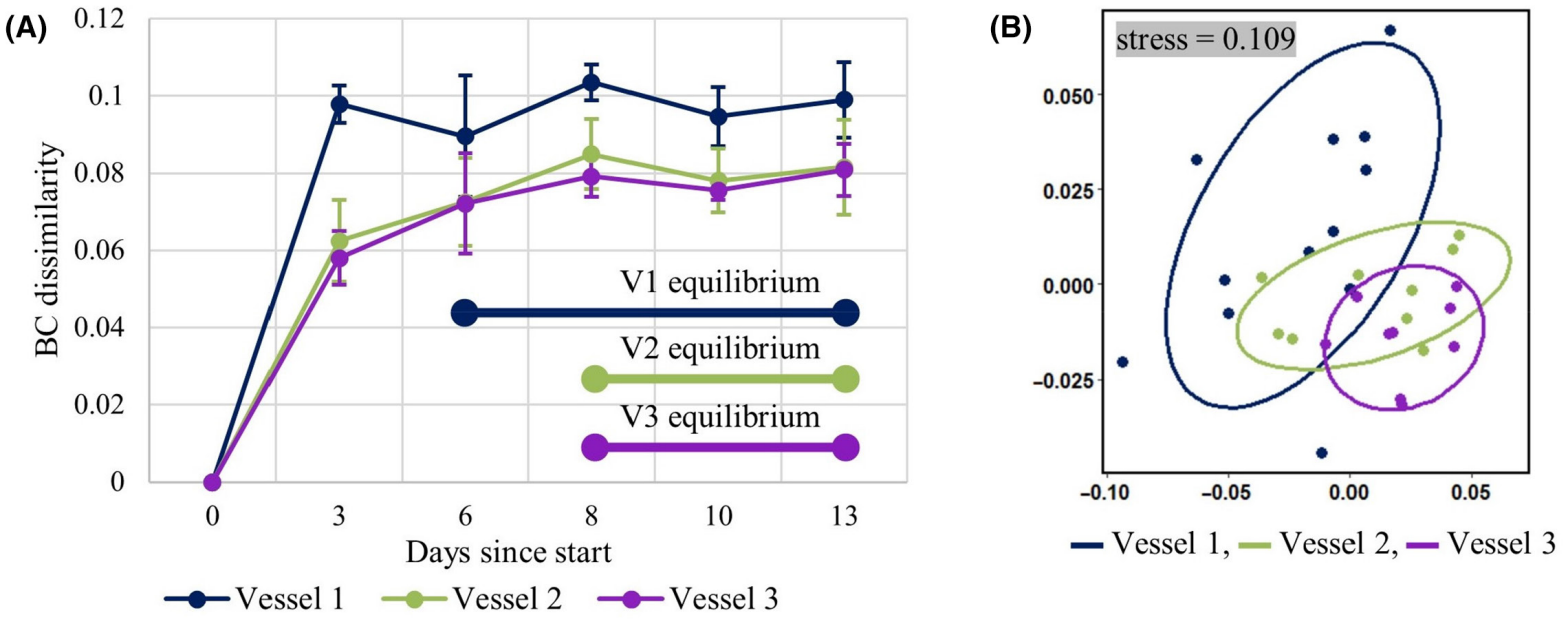
(A) The BC dissimilarity for each MiGut vessel, averaged across all three models, during the equilibration period; (B) an NMDS plot of the equilibria of each vessel—each point indicates a sample from the corresponding MiGut vessel (across all models).

**FIGURE 6:**
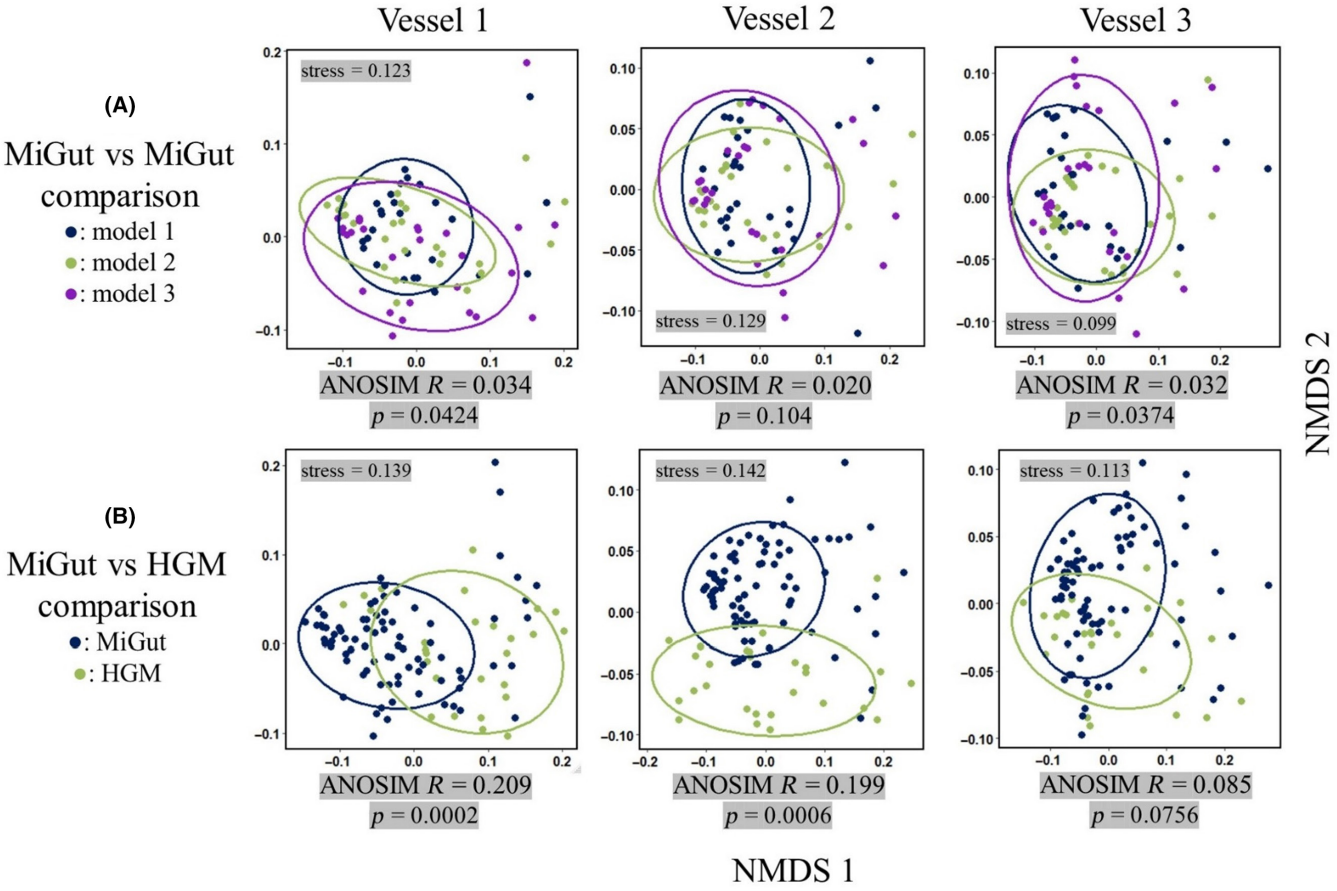
Inter‐model comparisons visualised with NMDS plots (calculated using Bray–Curtis dissimilarity matrices). (A) Comparison between MiGut models, (B) comparison between MiGut and HGM.

### Data analysis

Non‐metric multidimensional scaling of the Bray–Curtis dissimilarity matrix (NMDS) (Legendre & Legendre, 2012) was used to represent the data in 2D plots, as it preserves rank orders of dissimilarities. The fit of the NMDS ordination is evaluated with “stress” where stress <0.2 is typically considered an acceptable representation (Clarke, 1993). Analysis of similarities (ANOSIM) tests were used to evaluate community similarity. ANOSIM compares the rank similarities of samples within and between groups, generating an R statistic which varies from 1 (similarities within a group are greater than similarities between a group, i.e. groups are dissimilar) to 0 (replicates within and between groups have similar rank dissimilarities, i.e. groups are similar) (Clarke, 1993). Statistical significance is determined by comparing R to its null distribution, calculated from 9999 random permutations. A value of *p* < 0.05 is generally considered statistically significant, while a higher value means the null hypothesis (there is no difference between the microbial communities of the groups) cannot be rejected. Note that when R is close to zero, *p* is often greater than 0.05. Both these results have the same outcome (populations are similar); thus, in this analysis, the R statistic is often more important that the *p* value. Finally, BC dissimilarity is an ecological measure of dissimilarity between two groups ranging from 0 (identical) to 1 (completely dissimilar) (Ricotta & Podani, 2017). NMDS plots, ANOSIM tests and BC dissimilarities were all calculated with RStudio (RStudio: Integrated Development Environment for R, version 2021.9.2.382, www.rstudio.com). Heatmaps  and PMCCs were calculated with Microsoft Excel (Microsoft Corporation, version 2021).

## RESULTS

### Development of novel platform

An iterative design cycle was used to develop MiGut, with outcomes judged against the performance of a well‐validated, clinically reflective HGM (Moura et al., 2019; Normington et al., 2021). The engineering process focused on (i) miniaturising the system to reduce bench space; (ii) developing a user‐centric design for easier model set‐up and operation; (iii) incorporating the use of Internet of Things technology to stream data in real‐time for improved process monitoring and control.

The novel design of MiGut is shown in Figure 2. Each MiGut platform consists of four triple‐stage models and within each model, there are three vessels representing the proximal (V1), medial (V2) and distal (V3) colon, with each vessel containing a much lower fluid volume (45 mL) compared to the HGM (300 mL). The MiGut platform also has a controller, a pumping unit and a media feed. The internal geometry of each reactor is specifically designed to maintain proper fluid flow direction and promote mixing through the continuous bubbling of nitrogen, which also maintains an anaerobic environment. Model temperature is maintained with heat mats attached to the back of the reactors and pH is controlled in each vessel (5.5 ± 0.1, 6.25 ± 0.1, 6.75 ± 0.1 in V1, V2 and V3) by selectively adding 0.1 M HCl or 0.1 M NaOH. This is done with a single pumping unit, which uses 12‐way flow selector valves to direct the flow as required, minimising the amount of ancillary equipment. Temperature and pH were maintained effectively within the required limits throughout the study, with the controller quickly responding to any fluctuations (Supporting Information, Section 1).

**FIGURE 7:**
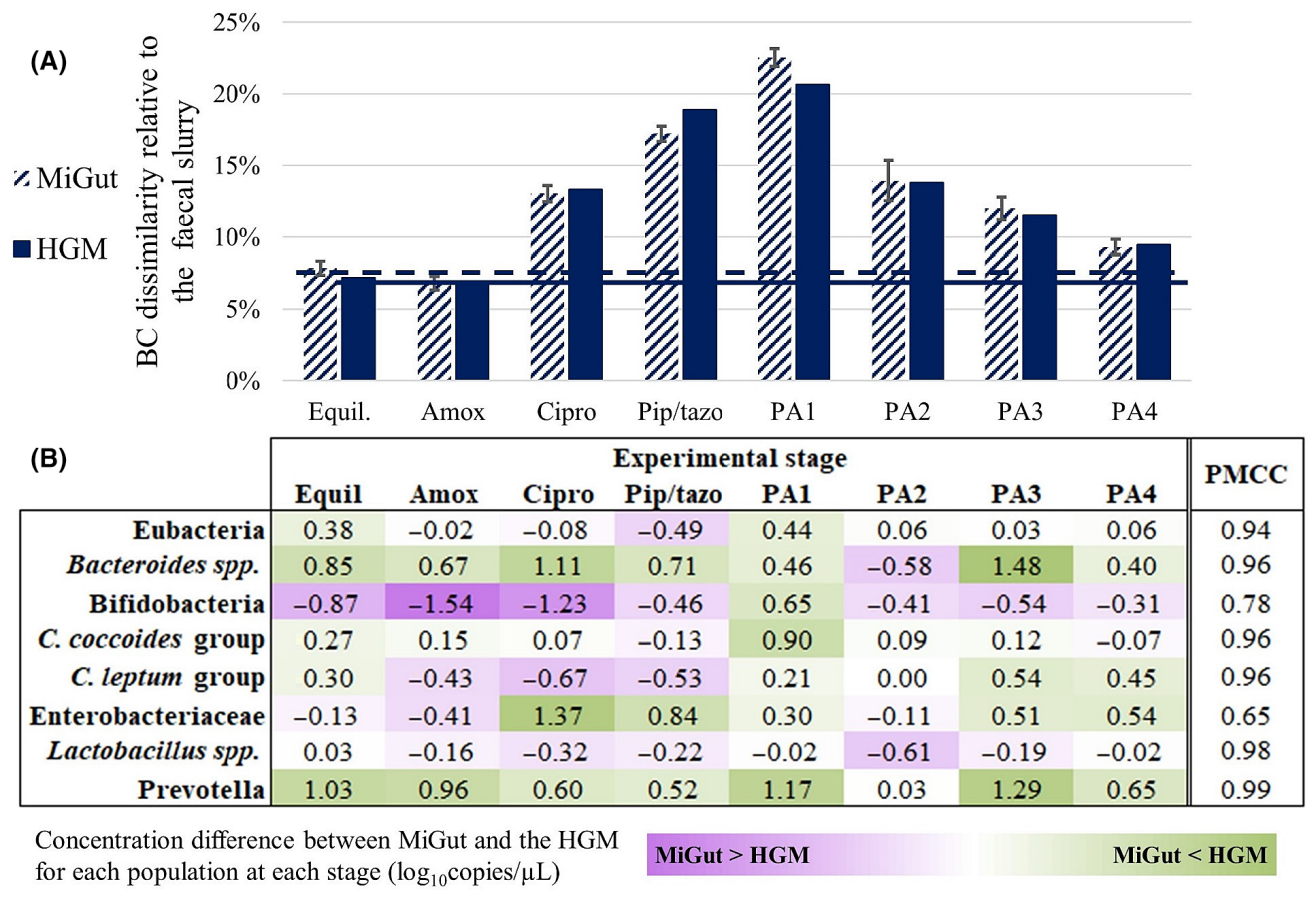
(A) Bray–Curtis dissimilarity relative to the faecal slurry for V3 of both MiGut and the HGM throughout the study—horizontal lines show the equilibrium value; (B) heatmap showing the difference (in log_10_copies/μL) between MiGut and the HGM for each population at each stage—purple cells indicate MiGut > HGM, green cells indicate HGM > MiGut. Additionally, the product‐moment correlation coefficient was calculated between MiGut and the HGM for each population.

V1 receives a continuous flow of nutrient‐rich media (see Section 2) at a flow rate of 0.045 mL/min to simulate a 48 h retention time (within the whole model), with other vessels connected sequentially (shown in Figure 2F). Together with the varying pH, the nutrient availability gradient drives differentiation between the vessels and establishes complex trophic chains, a key benefit of running multi‐stage models. Overall, each MiGut model has a reduced footprint and can easily be assembled and operated, facilitating future developments in automation and scale‐up.

All models are monitored and controlled from a central unit, with key data (pH, temperature, acid/alkali additions) being continually streamed to a database (Microsoft Azure) for processing and visualisation, allowing for real‐time monitoring. Vessels are periodically sampled and key bacterial populations are monitored by rtPCR, which has been shown to correlate well with bacterial enumeration data (Moura et al., 2020). An overview of the workflow is shown in Figure 3.

**FIGURE 8:**
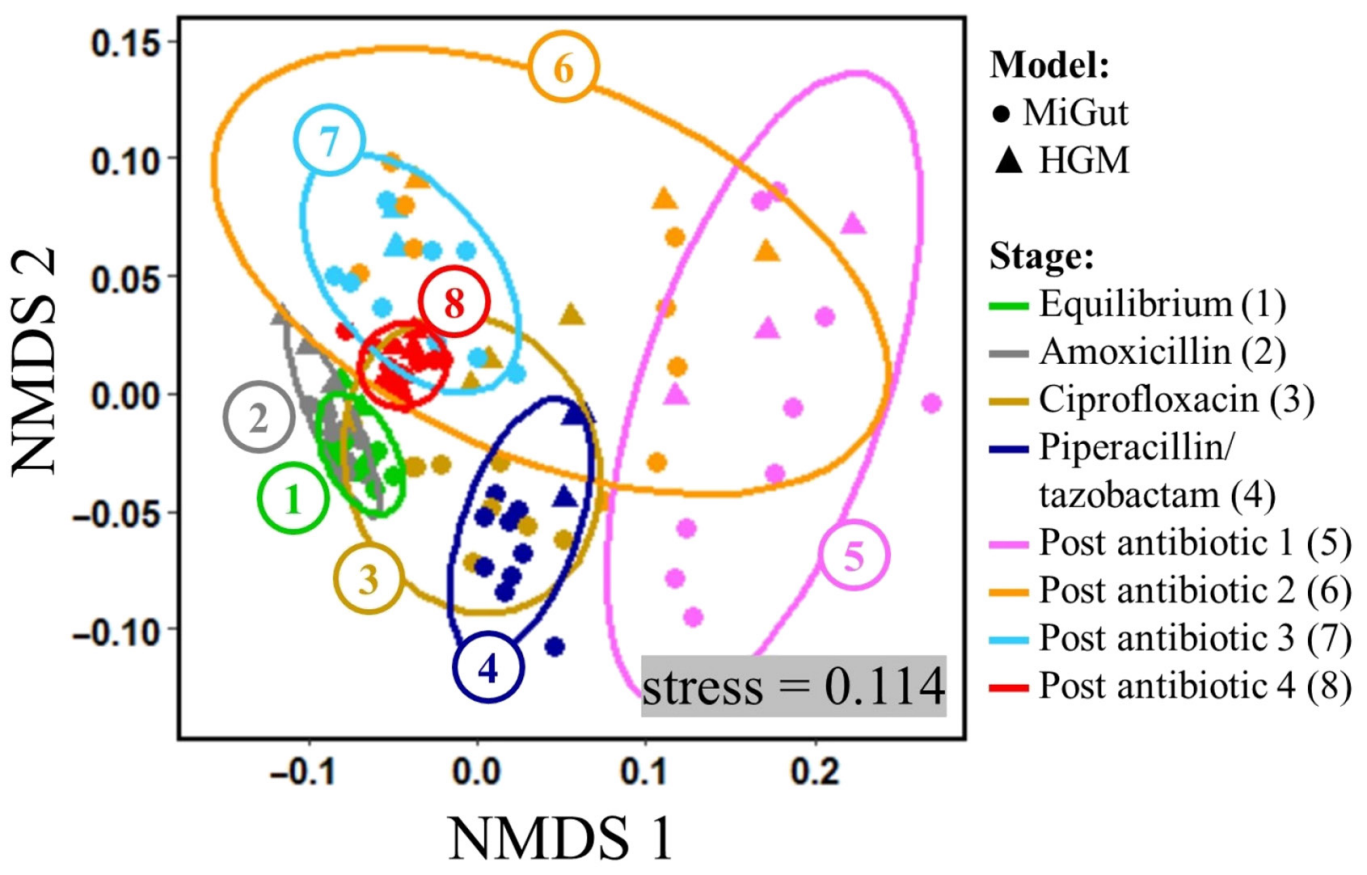
NMDS ordination for the whole study (calculated using Bray–Curtis dissimilarity matrix). Each point indicates a sample taken from a single reactor, with circles representing MiGut models and triangles representing the HGM. Ellipses and colours show grouping by stage of the experiment, from equilibrium through dosing, to post‐antibiotic. Closer groupings of points at each stage indicate less variability between samples. Groups which overlap have greater similarity than groups which are separate.

### MiGut models recapture the bacterial composition of the human donor material

For the study of the human microbiome in vitro, it is important that MiGut models can accurately reproduce the population characteristics of the faecal slurry with which they are seeded. It has been previously shown that the HGM effectively recaptures the microbial composition of multiple donors (Buckley, Moura, Arai, et al., 2021); in order to confirm a similar reliability of the MiGut platform, a preliminary study was conducted whereby four different faecal slurries were used to inoculate up to four MiGut models (exact number of replicates for each condition was based on the laboratory capacity at the time). The models were run under equilibration conditions for 2 weeks, after which samples were taken from V3 for sequencing and taxonomic analysis. Analyses from in vitro gut models most commonly focus on V3 as it represents the conditions of the distal colon and so most closely resembles the composition of human faecal samples, allowing for comparison with clinical studies. Accordingly, V3 is considered to be the most relevant when modelling gastrointestinal infections (e.g. *C. difficile* infection) (Moura et al., 2019; Oldfield IV et al., 2014) and is the vessel used for this analysis.

A principal coordinate analysis (PCoA) was performed using 16S rRNA sequencing data (Figure 4). The resulting plot shows that, in each experiment, the MiGut V3 contents cluster closely to the initial faecal inoculum. Taxonomic analysis shows that the majority of the main taxa present in the faecal slurry are also present in the models; thus, MiGut effectively recaptures the initial microbial composition.

### The communities formed in MiGut are stable, reproducible and correlate with a clinically relevant HGM

The MiGut platform was then tested over a 9‐week period alongside a HGM under identical conditions, allowing for direct comparisons. Initially, the microbial populations within the models were allowed to equilibrate for 2 weeks. Then, they were exposed to a series of three antibiotics for 1 week each and monitored for a further 4 weeks after dosing was finished. Due to a technical fault in the initial week, one MiGut model was stopped; thus, the data reported are from the remaining three models.

Bacterial populations in all models were monitored thrice weekly by quantitative (rtPCR) of key communities. These groups were specifically selected as key populations representative of microbial homeostasis in human gut, and they are therefore a good indicator of antibiotic impact on the GM (Moura et al., 2020). Furthermore, historical studies across the 30‐year history of triple‐stage fermentation models have often monitored these same populations, albeit with culture‐based methods; therefore, there is an overwhelming amount of historical data regarding population dynamics.

The initial 2‐week equilibration stage allows bacterial populations to stabilise in the gut reactor environment, conferring functional properties such as colonisation resistance to pathogens. It is often challenging to characterise this community stability: in the HGM, it has been shown that bacterial populations reach an equilibrium by the end of a 2‐week period (Buckley, Moura, Arai, et al., 2021). This was also observed in the MiGut models by plotting the Bray–Curtis (BC) dissimilarity relative to the faecal inoculum (Figure 5A). The gradient of each line (calculated in three‐point sliding windows) indicates the rate of change of composition of the microbial communities. All vessels initially had a high rate of change, which plateaued as populations reached an equilibrium (defined here by gradient <0.002/day). Based on this analysis, V1 equilibrated on Day 6, while V2 and V3 equilibrated on Day 8. Upon reaching equilibrium, V1 showed the greatest level of dissimilarity to the faecal inoculum, compared with V2 and V3. This is reflective of the microbial populations found within the different sites of the proximal colon (reflected in V1) and the distal colon (reflected in V3). Analysis of similarity (ANOSIM, see Section 2) tests further showed that the equilibria achieved in V2 and V3 were very similar (ANOSIM statistic *R* < 0.1, *p* = 0.125) while the V1 equilibrium had a greater difference from V2 and V3 (*R* = 0.129, *p* = 0.055 and *R* = 0.452, *p* < 0.001, respectively). These differences can be visualised by using non‐metric multidimensional scaling (NMDS, Figure 5B), where V1 forms a cluster which is largely distinct from those of V2 and V3. Furthermore, the variability in values during equilibrium decreased from V1 through to V3. This is reflected in the standard deviation of the BC dissimilarity (Table 1) and in the decreasing spread of each group in the NMDS plot.

**TABLE 1:** BC dissimilarity data for both the MiGut platform (averaged across all models) and the HGM.

	BC dissimilarity from faecal inoculum at equilibrium		Standard deviation
	HGM	MiGut (%)	MiGut (%)
V1	N/A	9.67	1.61
V2	9.68%	8.15	1.53
V3	7.18%	7.85	0.81

The same techniques were used to define equilibrium in the HGM (Supporting Information Section 2); based on this analysis, the populations in V1 of the HGM did not equilibrate. However, V2 and V3 both equilibrated on Day 10, with BC dissimilarities close to those achieved with MiGut (Table 1). ANOSIM tests were further used to evaluate inter‐model similarity (i) between MiGut models, and (ii) between the MiGut platform and the HGM. Replicate MiGut models showed high degrees of similarity across all three vessels (*R* < 0.1, Figure 6A). When comparing MiGut with the HGM (Figure 6B), V1 and V2 showed high similarity with some differences (0.1 < *R* < 0.25), while V3 was highly similar (*R* < 0.1). Overall, V3 showed the greatest similarity between MiGut and the HGM.

### Antibiotic dosing severely disrupts the microbiome, with MiGut closely matching results from a clinically relevant HGM

As discussed previously, V3 represents the distal colon, and thus, its content is the most closely related to human faecal samples. Consequently, we also focused on this vessel when reporting the effects of antibiotic dosing on the residing populations.

BC dissimilarities were calculated relative to the faecal slurry across all stages of the experiment using data from rtPCR. The results, shown in Figure 7A, indicate that antibiotic dosing disrupted the microbiome from its initial equilibrium, leading to a composition that was overall more dissimilar from the faecal slurry. In the subsequent post‐antibiotic stages, the microbiome showed signs of recovery as BC dissimilarity once again approached the equilibrium value. However, neither MiGut or the HGM fully returned to its initial equilibrium, showing the long‐term effects of antibiotic dosing. Importantly, the results from MiGut and the HGM correlated extremely well with one another (PMCC > 0.98), with BC dissimilarities being almost identical throughout. The differences in the concentrations of populations between models are explored in detail in Figure 7B. Across the study, the biggest deviation was observed with *Bifidobacteria*, where the abundance in MiGut was 1.54 log_10_copies/μL higher than in the HGM. However, this difference subsequently reduced to only 0.31 log_10_copies/μL as the experiment proceeded. Similar trends were observed in other populations: *C. leptum*, Enterobacteriaceae, and *Lactobacillus* spp., for example, all show increased dissimilarity during the antibiotic dosing stages. Overall, post‐antibiotic Week 4 is the stage where MiGut and the HGM are most similar, with a maximum deviation of 0.65 log_10_copies/μL, also reflected in Figure 7A. Additionally, the product‐moment correlation coefficient (PMCC) was calculated for each population to quantify how well a change in population in the HGM is replicated in the MiGut models. Most populations had strong, positive, correlations indicating that changes were consistent between both systems. The lowest PMCC was that of Enterobacteriaceae (PMCC = 0.65), suggesting some differences in the antibiotic response between the HGM and MiGut for that population. *Prevotella* spp., which had a higher difference in concentration between the models throughout, had the highest PMCC (0.99).

Plotting the data on an NMDS plot and grouping by stage (Figure 8) shows that during antibiotic dosing there were pronounced changes in the GM composition as the groups moved away from the equilibrium region (Group 1). This analysis also showed that when the models were challenged with antibiotics, variation increased, reflected by a wider spread of data points. In the post‐antibiotic period, when populations were allowed to recover, the inter‐ and intra‐model variation decreased, and the microbiome composition moved back towards the initial equilibrium. Importantly, the samples in the final post‐antibiotic stage (Group 8) formed a closely packed group, which was distinct from that of the initial equilibrium (ANOSIM *R* = 0.92, *p* = 0.0001), indicating that even 4 weeks after antibiotic withdrawal the bacterial populations did not return to their original state. Within the stage groupings, points corresponding to MiGut and the HGM occupied the same space, showing that there was no difference between the two sets of models, further supporting the equivalence of MiGut to the HGM.

## DISCUSSION

The use of in vitro models that aim to simulate the human GM offers a valuable insight into how multiple internal and external factors can interact to affect the microbial populations. However, the inherent complexity of these models required to closely mimic the colonic environment has previously meant that their scale‐up is impractical (Sardelli et al., 2021). MiGut has been designed to address these shortcomings by carefully considering the limiting factors of current technologies and addressing them through an engineering design process, while remaining reflective of the in vivo environment. The MiGut platform greatly reduces bench space, set‐up time, and user intervention when compared to other triple‐stage gut models. In doing so, MiGut can easily be scaled up to include multiple sets of models running simultaneously in a resource‐efficient way. This combination of scalability, ease of use, and process control makes MiGut unique in its field.

Initially, a pilot study evaluated how well the MiGut models recaptured the microbial communities of faecal inocula using 16S rRNA sequencing. Four different runs were performed, and for each of the faecal slurries used, there were four distinct clusters on the principal coordinate plot (Figure 4). This indicates that the equilibria achieved by the MiGut models accurately recaptured the original faecal slurry and, for the most part, preserved the differences between each of the starting points. This was underlined by comparing the taxonomic compositions of the slurries with that of the MiGut models, which shows that they recaptured many of the taxa present in the faecal slurry. Additionally, the close clustering of different models further demonstrates reproducibility between individual MiGut models.

The MiGut platform was then tested in a lengthy and demanding validation study, running for 9 weeks continuously. Not only did this allow for evaluation of the populations at equilibrium but also the response to antibiotics was investigated and compared directly to a clinically relevant and commonly used HGM. During the initial equilibration period, it was important to demonstrate that the bacterial populations were stable across all models. It was found that communities equilibrated within 6 (V1) and 8 (V2 and V3) days of inoculation, which is consistent with reports from other gut model systems (Auchtung et al., 2015). The equilibria reached were distinct in V1 compared with V2 and V3, highlighting the advantages of a multi‐stage system which can simulate different colonic regions. Variability both within and between MiGut models during equilibrium was low and decreased from V1 through to V3 (Figure 5A; Table 1). Furthermore, V1 was more dissimilar to the faecal slurry when compared with V2 and V3. This is to be expected since V3 represents the distal colon, and its composition is therefore the most comparable with faecal samples. Overall, dissimilarities between the faecal slurry and the equilibria were at most 10% (V1) and 8% (V2 and V3), suggesting there is little deviation from the slurry. The use of ANOSIM tests (Figure 6A) further demonstrated that the MiGut models showed good repeatability, with only small differences observed between models.

A single HGM was run alongside the MiGut platform under identical conditions to evaluate the consistency of the new model design compared with the original. Based on the analyses used, V1 of the HGM did not equilibrate in the initial stages and showed a higher degree of dissimilarity between MiGut and the HGM compared with the other vessels (Figure 6B and Supporting Information Section 2). However, we believe this was due to experimental artefact, where pH and volume fluctuations caused some variation of the conditions in V1 of the HGM, influencing the microbial populations. Meanwhile, V2 and V3 showed high degrees of similarity between the two model systems (Figure 6B). Although there were no replicates of the HGM due to practicality and cost, it has previously been shown that results are consistent across multiple HGMs (Moura et al., 2019; Normington et al., 2021).

Antibiotic dosing as outlined in Table 2 had a profound effect on the GM in V3. Although amoxicillin had a less pronounced impact, subsequent antibiotics caused dramatic changes to the bacterial populations, leading to a large deviation from the established equilibrium (Figure 7A). Generally, the concentrations of the measured populations were similar between the HGM and MiGut throughout: *C.coccoides*, *C. leptum*, and *Lactobacillus* spp. had maximum differences of 0.90, 0.67, and 0.61 log_10_copies/μL, respectively. Other populations (*Bifidobacteria*, Enterobacteriaceae, *Prevotella spp.*) showed more variation. However, by the final week of the study, differences in concentrations for each bacterial population had reduced to at most 0.65 log_10_copies/μL, indicating that the microbiomes in different models equilibrate to the same levels if undisturbed. The PMCC gives additional insight into how well population changes in the HGM were reflected in MiGut: overall, populations correlated extremely well, with six out of the eight reported populations having PMCC > 0.9. *Bifidobacteria* and Enterobacteriaceae showed some differences (PMCC of 0.78 and 0.65, respectively), mostly during the antibiotic dosing. A greater degree of variability is expected during dosing as the populations are subject to external stressors. Interestingly, *Prevotella* spp., which had a relatively high concentration difference between MiGut and the HGM throughout, had a PMCC of 0.99: indicating that although there were absolute differences in concentration, the relative changes from one stage to another in the HGM were precisely replicated within MiGut. However, our data suggest that even with a prolonged recovery period, the bacterial populations fail to return to their original equilibrium in both the MiGut and the HGM (Figures 7A and 8), demonstrating the long‐term effects these medications can have.

**TABLE 2:** Outline of the 9‐week validation study of MiGut.

Week	1	2	3	4	5	6	7	8	9
Stage	E1	E2	Amoxicillin	Ciprofloxacin	Piperacillin/tazobactam	PA1	PA2	PA3	PA4
	Equilibration		Antibiotic dosing			Post‐antibiotic			

In general, in vitro models (including MiGut) offer a very useful tool for simulating the GM. However, a criticism of these systems is that they can be over‐simplistic and fail to adequately simulate some of the complexity of the in vivo environment (Ewin et al., 2023). For example, it has been shown that there are differences between the sessile and planktonic bacterial communities of the colon, and the ability to sample both groups would give a more complete view of the GM (Bircher et al., 2020; Crowther et al., 2016). There is also the potential for build‐up of metabolic products which would normally be absorbed by the host, which can artificially change the in vitro environment. Furthermore, although these models are suitable for exploring microbe‐microbe or microbe‐drug interactions, they lack the capacity to study how these changes affect the host physiology. Simulating these interactions in vitro is particularly challenging due to the different culture requirements of prokaryotic and eukaryotic cells (Sardelli et al., 2021). This has been addressed by a number of recent systems which sustain the required oxygen gradient and media perfusion to co‐culture human and bacterial cells (Jalili‐Firoozinezhad et al., 2019; Zhang et al., 2021). By combining such technologies with MiGut, it is hoped that a more complete in vitro simulation of the microbiome can be achieved in the future. Finally, the results reported in this paper focus only on the bacterial populations, but the GM is a complex community that includes also fungi, viruses, archaea, protozoa (Cao et al., 2022; Vemuri et al., 2020; Zhang et al., 2022). The complete analysis of such a complex environment is difficult and often impractical; however, it will be important for future works to consider how all these groups interact within the system.

Overall, extensive and rigorous testing of the MiGut platform has demonstrated that it is capable of supporting complex bacterial communities. Taxonomic analysis of different assays showed that MiGut can recapture the nuclear composition of the faecal slurry used to initiate an experiment. These communities readily stabilise within the vessels, with excellent repeatability between models and good similarity with a clinically relevant HGM. Furthermore, the response to multiple external stressors (i.e. antibiotics) was consistent across all replicates, correlating extremely well with the benchmark system and demonstrating the suitability of MiGut to simulate both healthy and perturbed states of the human GM.

In summary, the MiGut platform has miniaturised and improved current triple‐stage gut modelling methods, offering far greater scalability. This technology has great potential to improve the reliability of results and allow for far more complex studies. The composition of the human GM can be affected by multiple factors (e.g. age, ethnicity, diet, pathogen exposure or medication) which, in turn, affect clinical outcomes (Kho & Lal, 2018). The throughput and scalability of MiGut is necessary to allow investigation of several interacting factors simultaneously, with the support of biological replicates. This will lead to a greater understanding of the cause–effect relationships between xenobiotics and the intestinal microbiome, ultimately informing clinical practice and, we anticipate, leading to more targeted and effective healthcare.

## AUTHOR CONTRIBUTIONS

**William A. Davis Birch:** Conceptualization (equal); formal analysis (lead); investigation (lead); visualization (lead); writing—original draft (lead). **Ines B. Moura:** Conceptualization (equal); data curation (equal); investigation (equal); methodology (equal); supervision (equal); writing—review and editing (equal). **Duncan J. Ewin:** Investigation (equal); methodology (equal); writing—review and editing (supporting). **Mark H. Wilcox:** Conceptualization (supporting); writing—review and editing (supporting). **Anthony M. Buckley:** Conceptualization (equal); supervision (equal); writing—review and editing (equal). **Peter R. Culmer:** Conceptualization (equal); supervision (equal); writing—review and editing (equal). **Nikil Kapur:** Conceptualization (equal); project administration (equal); supervision (equal); writing—review and editing (equal).

## FUNDING INFORMATION

No funding information provided.

## CONFLICT OF INTEREST STATEMENT

IM has received funding to attend conferences from TECHLAB Inc. MW has received honoraria for consultancy work, financial support to attend meetings and research funding from Astellas, AstraZeneca, Abbott, Actelion, Alere, Bayer, bioMérieux, Cerexa, Cubist, Da Volterra, Durata, European Tissue Symposium, Merck, Nabriva Therapeutics plc, Pfizer, Qiagen, Roche, Seres Therapeutics Inc., Synthetic Biologics, Summit, and The Medicines Company. AB has received financial support to attend meetings and research funding from Seres Therapeutics Inc., Motif Biosciences plc., Nabriva Therapeutics plc, Tetraphase Pharmaceuticals, Almirall SA, GlaxoSmithKline plc, and Hayashibara Co. Ltd. All other authors state no conflict of interest.

## ETHICS STATEMENT

The collection and use of human faeces in our gut model have been approved by the School of Medicine Research Ethics Committee, University of Leeds (MREC 15‐070—Investigation of the Interplay between Commensal Intestinal Organisms and Pathogenic Bacteria). Participants were provided with a “Participant Information Sheet” (PIS) detailing a lay summary of the in vitro gut model and the scientific work they were contributing to by providing a faecal donation. Within this PIS, it is explained that by providing the sample, the participant is giving informed consent for that sample to be used in the gut model.

## Supporting information


Supporting Information S1.

## Data Availability

Additional rtPCR data are available from the University of Leeds at https://doi.org/10.5518/1166. Bacterial taxonomic data used for Figure 4 are available on the Sequence Read Archive under BioProject: PRJNA930754.
