# Supplementary material for: MiGut: A scalable in vitro platform for simulating the human gut microbiome—Development, validation and simulation of antibiotic‐induced dysbiosis
Source: Microb Biotechnol. 2023 Apr 10;16(6):1312–24. doi: 10.1111/1751-7915.14259 (PMC10221534; doi:10.1111/1751-7915.14259)
Supplement: Supplementary file 1 — Supporting Information S1. [file MBT2-16-1312-s001.docx]

# Results: pH and temperature plots for MiGut at steady state


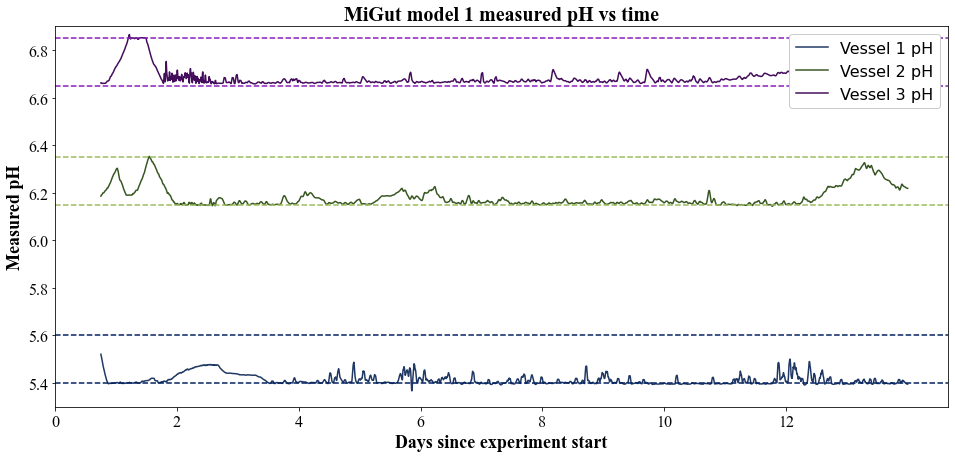


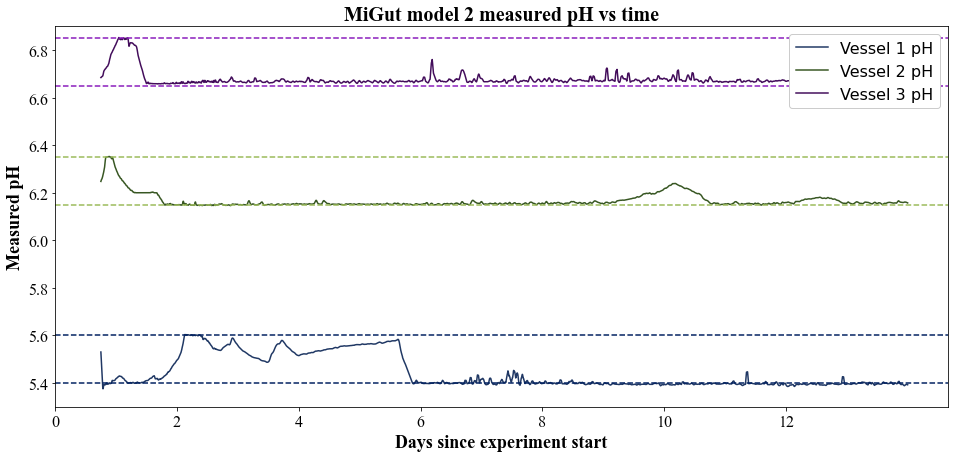


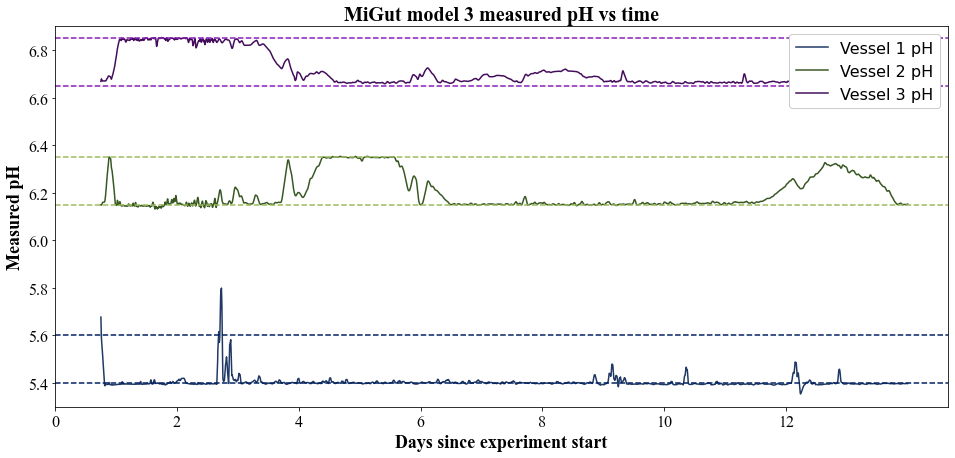


Figure 1: pH vs time plots for each of the MiGut reactors during the equilibration stage of the experiment. Measured pH for V1, V2, and V3 are shown in blue, green, and purple lines, respectively. Dotted lines show the upper and lower limits for the target pH of each vessel.


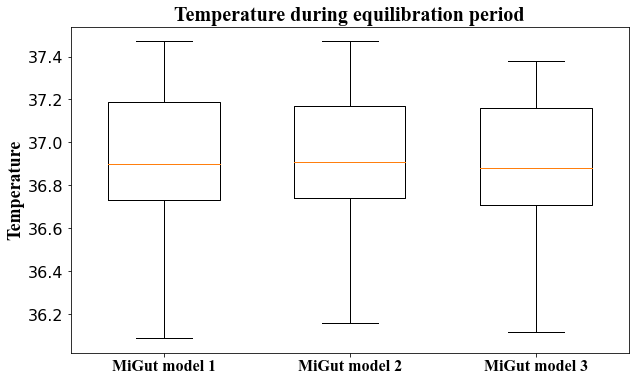


Figure 2: Box and whisker plots for the temperature in each of the three MiGut models. Note that temperature is measured in all vessels – the plotted temperature is the maximum of the three.

# Results: Results of the standard triple-stage human gut model

Full Bray-Curtis dissimilarity results are available from the University of Leeds at https://doi.org/10.5518/1166


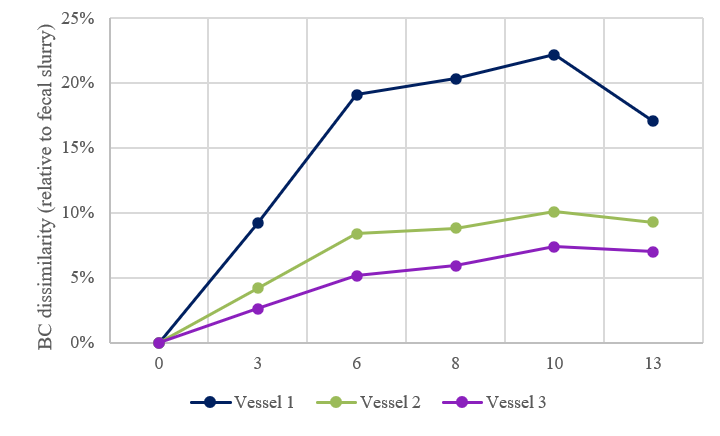


Figure 3: Bray-Curtis dissimilarities from the fecal inoculum for the human gut model during equilibration

# Methods: Setup of triple-stage human gut model


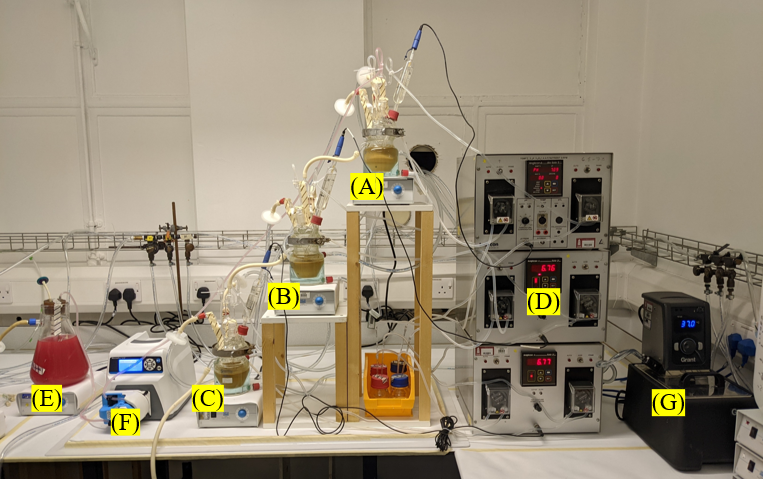


Figure 4: Typical setup of a triple-stage human gut model. (A) Vessel 1, (B) Vessel 2, (C) Vessel 3, (D) pH controllers (one for each vessel), (E) Nutrient-rich media, (F) Peristaltic pump for media, (G) Heated water pump

- Vessel 1 (proximal colon): high nutrient availability, pH 5.4 – 5.6, 280ml
- Vessel 2 (medial colon): low nutrient availability, pH 6.15 – 6.35, 300ml
- Vessel 3 (distal colon): low nutrient availability, pH 6.65 – 6.85, 300ml

Vessel 1 is top fed with a complex growth medium detailed in Table 1 at a constant rate of 0.3 ml/min (simulates a 48h retention time). The arrangement of the vessels means the outlet of Vessel 1 flows into Vessel 2 and so on, creating a nutrient availability gradient (from high to low). All vessels are maintained at body temperature by a water jacket, continuously stirred and continually sparged with N_2_ to maintain anaerobic conditions. This setup has been used extensively in the literature and has been historically validated against samples from sudden death victims (Macfarlane et al., 1998).

Table 1: Composition of the nutrient-rich media used in the study.

| Constituent | Final concentration | Supplier |
| --- | --- | --- |
| Magnesium sulphate | 0.01 g/L | Sigma |
| Calcium chloride | 0.01 g/L | Sigma |
| Sodium chloride | 0.1 g/L | Sigma |
| Di-potassium monohydrogen phosphate | 0.04 g/L | Fisher |
| Potassium di-hydrogen phosphate | 0.04 g/L | Fisher |
| Sodium hydrogen carbonate | 2.0 g/L | Sigma |
| Haemin | 0.005 / L | Sigma |
| Cysteine HCL | 0.5 g/L | Sigma |
| Bile Salts | 0.5 g/L | Sigma |
| Arabinogalactan | 1.0 g/L | Chem Cruz |
| Tween 80 | 2 mL / L | Sigma |
| Pectin | 2.0 g/L | Acros Organics |
| Starch | 3.0 g/L | Oxoid |
| Vitamin K1 | 10 µL / L | Sigma |
| Peptone Water | 2.0 g/L | Oxoid |
| Yeast extract | 2.0 g/L | Oxoid |
| Chenodeoxycholic acid | 0.25 mg/L | Sigma |
| Lithocholic acid | 0.017 mg/L | Sigma |
| Mucin | 2 g/L | Sigma |
| Glucose* | 0.4g/L | Sigma |
| Resazurin* | 0.005g/L | Sigma |

*****added after autoclaving through a sterile 0.2µm filter

| **Target** | **Primer** | **Sequence (5’-3’)** | **Product size (bp)** | **Annealing (time, temp)** |
| --- | --- | --- | --- | --- |
| Eubacteria | 8F | AGTTTGATCCTGGCTCAG | 417 | 45s, 54 ºC |
|  | 515R | GNATTACCGCGGCNGCTG |  |  |
| Probe | 338P | FAM GCTGCCTCCCGTAGGAGT BHQ1 |  |  |
| *Bacteroides* spp. | Bac303F | GAAGGTCCCCCACATTG | 419 | 45s, 54 ºC |
|  | Bac708R | CAATCGGAGTTCTTCGTG |  |  |
| *Bifidobacterium spp.* | Bif551F | CGCGTCNGGTGTGAAAG | 244 | 20s, 55ºC |
|  | Bif794R | CCCCACATCCAGCATCCA |  |  |
| *C. coccoides* group | Eub338F | ACTCCTACGGGAGGCAGC | 139 | 30s, 56 ºC |
|  | Erec482R | GCTTCTTAGTCANGTACCG |  |  |
| *C. leptum* group | Sg-clept-F | GCACAAGCAGTGGAGT | 241 | 45s, 54 ºC |
|  | Sg-clept-R | CTTCCTCCGTTTGTCAA |  |  |
| Enterobacteriaceae | Eco1457F | CATTGACGTTACCCGCAGAAGAAGC | 190 | 45s, 60 ºC |
|  | Eco1652 | CTCTACGAGACTCAAGCTTGC |  |  |
| *Lactobacillus spp.* | Lacto-F | GAGGCAGCAGTAGGGAATCTTC | 126 | 45s, 60 ºC |
|  | Lacto-R | GGCCAGTTACTACCTCTATCCTTCTTC |  |  |
| *Prevotella spp.* | CFB286F | GTAGGGGTTCTGAGAGGA | 446 | 30s, 56 ºC |
|  | CFB719R | AGCTGCCTTCGCAATCGG |  |  |

# Methods: PCR methodology and primers

Table 2: Primers used for real-time PCR analysis

# Methods: 16S rRNA gene sequencing and taxonomic analysis

DNA quality and double-stranded quantities were determined using the picogreeen absorption method. Bacterial 16S rRNA V4 fragments were PCR amplified using NEBNext Q5 Hot Start HiFi PCR master mix (NEB, U.K.) with universal 16S rRNA V4 primers [564F (TCGTCGGCAGCGTCAGAT GTGTATA>AGAGACAG-AYTGGGYDTAAAGNG) and 806R (GTCTCGTGGGCTCGGAGATGTGTATAAGAGACAGTACNVGGGTATCTAATCC)] with Illumina adaptor sequence overhangs included using the PCR cycle [denaturation (95°C x3 min for 1 cycle), amplification (95°Cx30 sec, 50°Cx30 sec, 72°Cx30 sec, for 28 cycles) and final elongation (72°C x5 min for 1 cycle)]. PCR products were cleaned using AxyPrep Magnetic beads (Axygen, U.K.) and the 16S rRNA fragments checked using gel electrophoresis on an Agilent 2200 TapeStation system (Agilent Genomics, U.K.) before running a PCR for addition of the index sequences [denaturation (95°C x3 min for 1 cycle), amplification (95°C x30 sec, 55°C x30 sec, 72°C x30 sec, for 8 cycles) and final elongation (72°C x5min for 1 cycle)]. The fragments were cleaned, quantified (as before), normalised and samples sequenced on a MiSeq (Illumina) using a 2x250 bp paired-end reads cycle. Library preparation and sequencing was done at the University of Leeds sequencing facility.

Demultiplexed FASTQ files of 16S rRNA sequences were trimmed of adapter sequences using cutadapt (Martin, 2011), and samples were filtered based on the number of reads across all samples to provide similar coverage. We followed the standard operating procedure from the MOTHUR package (v.1.41.3) (Schloss et al., 2009). The paired reads were joined together and assembled into the contigs, and quality controlled based on the parameters such as maxambig=0, minlength=177 and maxlength=237. Unique sequences were aligned against a tailor-made reference generated from SILVA SEED database (version 132) and further filtered according to their start and end positions in the alignments. In order to reduce the possible redundancy to a minimum, the identical and very similar sequences (within 2bp mismatch) were merged. The chimeric sequences were discarded based on the built-in VSEARCH method (Rognes et al., 2016). OTUs (operational taxonomic units) were identified by clustering (0.5 UniFrac distance) the sequences and were assigned the consensus taxonomy information with label=0.03. Low abundant reads (<50 reads per sample) were removed before further analysis; however, this precludes the contribution of low abundance taxonomic families in our analysis whilst ensuring accuracy from sequencing artifact. Taxonomic analysis is represented as mean percent abundance from four technical replicates. The distance matrix based on thetayc approach was used for Principal Coordinates (PCoA) analysis and visualization for each group of samples, based on four technical replicates.

# References

Macfarlane, G. T., Macfarlane, S. & Gibson, G. R. (1998) Validation of a Three-Stage Compound Continuous Culture System for Investigating the Effect of Retention Time on the Ecology and Metabolism of Bacteria in the Human Colon. *Microbioal Ecelogy,* **35,** 180-187.

Martin, M. (2011) Cutadapt removes adapter sequences from high-throughput sequencing reads. *EMBnet.journal,* **17,** 10.

Moura, I. B., Normington, C., Ewin, D., Clark, E., Wilcox, M. H., Buckley, A. M. & Chilton, C. H. J. B. M. (2020) Method comparison for the direct enumeration of bacterial species using a chemostat model of the human colon. *BMC microbiology,* **20,** 1-12.

Rognes, T., Flouri, T., Nichols, B., Quince, C. & Mahe, F. (2016) VSEARCH: a versatile open source tool for metagenomics. *PeerJ,* **4,** e2584.

Schloss, P. D., Westcott, S. L., Ryabin, T., Hall, J. R., Hartmann, M., Hollister, E. B., et al. (2009) Introducing mothur: open-source, platform-independent, community-supported software for describing and comparing microbial communities. *Appl Environ Microbiol,* **75,** 7537-7541.
